# Contributors to self-report motor function after anterior cruciate ligament reconstruction

**DOI:** 10.1038/s41598-023-30291-x

**Published:** 2023-02-22

**Authors:** Daniel Niederer, Natalie Mengis, Max Wießmeier, Matthias Keller, Wolf Petersen, Andree Ellermann, Tobias Drenck, Christian Schoepp, Amelie Stöhr, Andreas Fischer, Andrea Achtnich, Raymond Best, Lucia Pinggera, Matthias Krause, Daniel Guenther, Maren Janko, Christoph Kittl, Turgay Efe, Karl-Friedrich Schüttler, Lutz Vogt, Michael Behringer, Thomas Stein

**Affiliations:** 1 Institute of Occupational, Social and Environmental Medicine, Department of Sports Medicine and Exercise Physiology, Goethe University Frankfurt, Ginnheimer Landstraße 39, 40487 Frankfurt, Germany; 2 Arcus Sportklinik, Pforzheim, Germany; 3 OSINSTITUT Ortho & Sport, Munich, Germany; 4 Klinik für Orthopädie und Unfallchirurgie, Berlin, Germany; 5 BG Klinik Hamburg, Hamburg, Germany; 6 Berufsgenossenschaftliche Unfallklinik Duisburg, Duisburg, Germany; 7 Orthopedic Surgery Munich, Munich, Germany; 8 Department of Orthopedic Surgery, Physical Medicine and Rehabilitation, University Hospital, Ludwig-Maximilians-University (LMU), Munich, Germany; 9 Department for Orthopaedic Sports Medicine, Klinikum Rechts der Isar, Munich, Germany; 10 Department of Orthopaedic and Trauma Surgery, Sportklinik Stuttgart, Stuttgart, Germany; 11 Department of Trauma, Hand and Reconstructive Surgery, University Medical Center Hamburg-Eppendorf, Hamburg, Germany; 12 Department of Orthopaedic Surgery, Trauma Surgery, and Sports Medicine, Cologne Merheim Medical Center, Witten/Herdecke University, Witten, Germany; 13 Department of Trauma, Hand, and Reconstructive Surgery, Goethe-University Frankfurt, Frankfurt, Germany; 14 Universitätsklinikum Münster, Munster, Germany; 15 Orthopaedicum Lich Giessen, Lich, Germany; 16 SPORTHOLOGICUM Frankfurt - Center for Sport and Joint Injuries, Frankfurt, Germany; 17 Department of Sport Sciences, Goethe University Frankfurt, Frankfurt, Germany

**Keywords:** Rehabilitation, Surgery

## Abstract

Numerous functional factors may interactively contribute to the course of self-report functional abilities after anterior cruciate ligament  (ACL)-reconstruction. This study purposes to identify these predictors using exploratory moderation-mediation models in a cohort study design. Adults with post unilateral ACL reconstruction (hamstring graft) status and who were aiming to return to their pre-injury type and level of sport were included. Our dependent variables were self-reported function, as assessed by the the KOOS subscales sport (SPORT), and activities of daily living (ADL). The independent variables assessed were the KOOS subscale pain and the time since reconstruction [days]. All other variables (sociodemographic, injury-, surgery-, rehabilitation-specific, kinesiophobia (Tampa Scale of Kinesiophobia), and the presence or absence of COVID-19-associated restrictions) were further considered as moderators, mediators, or co-variates. Data from 203 participants (mean 26 years, SD 5 years) were finally modelled. Total variance explanation was 59% (KOOS-SPORT) and 47% (KOOS-ADL). In the initial rehabilitation phase (< 2 weeks after reconstruction), pain was the strongest contributor to self-report function (KOOS-SPORT: coefficient: 0.89; 95%-confidence-interval: 0.51 to 1.2 / KOOS-ADL: 1.1; 0.95 to 1.3). In the early phase (2–6 weeks after reconstruction), time since reconstruction [days] was the major contributor (KOOS-SPORT: 1.1; 0.14 to 2.1 / KOOS-ADL: 1.2; 0.43 to 2.0). Starting with the mid-phases of the rehabilitation, self-report function was no longer explicitly impacted by one or more contributors. The amount of rehabilitation [minutes] is affected by COVID-19-associated restrictions (pre-versus-post: − 672; − 1264 to − 80 for SPORT / − 633; − 1222 to − 45 for ADL) and by the pre-injury activity scale (280; 103 to 455 / 264; 90 to 438). Other hypothesised contributors such as sex/gender or age were not found to mediate the time or pain, rehabilitation dose and self-report function triangle. When self-report function is rated after an ACL reconstruction, the rehabilitation phases (early, mid, late), the potentially COVID-19-associated rehabilitation limitations, and pain intensity should also be considered. As, for example, pain is the strongest contributor to function in the early rehabilitation phase, focussing on the value of the self-report function only may, consequently, not be sufficient to rate bias-free function.

## Introduction

A rupture of the anterior cruciate ligament (ACL) is a serious hazard for health and career advancement in sports^1^. Following ACL-reconstruction, affected athletes display a high risk for subsequent issues. Graft failure, contralateral ACL-injury^2,3^, or the development of osteoarthritis^4^ are often named in this context. These severe potential consequences highlight the importance of a rehabilitation process targeting a decrease in the risk for secondary health problems.

Beyond psychosocial readiness and morphological graft healing, particularly important targets of the rehabilitation process are the restoration of neuromuscular and motor knee-related function^5^. The Knee Injury and Osteoarthritis Outcome Score (KOOS) is a valid possibility to measure long and short-term patient-reported confidence in their knee-related function. Highlighted in a systematic review^5^, the KOOS possesses a re-injury-predictive value, in both isolation^6^ and as part of a testing battery^7–9^. Restoring functional abilities such as conidence in knee function following ACL-reconstruction may consequently lead to a decrease in the re-injury risk^10^. Monitoring self-report function as a potential surrogate of both functional abilities and self-confidence may be helpful in re-injury or osteoarthritis preventive measures and therapy settings.

Based on the individual courses of wound healing, functional abilities and psychological readiness, the time until rehabilitation completion and return to sports is variable^11^. Although it is not possible to define fixed time points at which a certain goal or functional ability is reached, time is, nevertheless, an important factor to consider^12^.

The individual wound healing progress, functional abilities, and psychological readiness do not only affect the time until rehabilitation completion, but also on the rehabilitation measures themselves. A sufficient training stimulus to reach functional progress and the risk for, inter alia, a graft failure must be weighted^13^. Therapy measures are dependent on the individual functional status^13^. The current functional status, in turn, has been found to be dependent on the pre-injury activity and pre-surgery functional status^13^. Another, contemporary contributor to the possibility to utilise exercises and trainings is the Coronavirus disease 2019 (COVID-19)-associated restrictions^14^. Herewith associated confinements cannot be neglected when therapy-associated function is rated.

Beyond the contributors highlighted so far, age, sex/gender^15,16^, pain intensity/perception during performance^17,18^, time passed between injury and reconstruction^19^ and kinesiophobia^20^ must also be taken into account when the function after ACL reconstruction is rated. Many of these numerous contributors are nested predictors of functional outcomes after anterior cruciate ligament reconstruction^21^.

From a theoretical point of view, a mediating effect of the amount of rehabilitation measures on how time since reconstruction affects functional abilities is possible. It is likely that this interaction differs further in the various post-reconstruction wound healing phases; the importance of pain, for example, decreases with increasing time. The different stages are, thus, of potential relevance as moderators of the interactions sketched above.

Conclusively, when the contribution of graft type or concomitant injuries^21^ are known in a study sample, a multitude of individual and spatiotemporal factors interact during the rehabilitation, return to sports and re-injury prevention processes following ACL reconstruction. We, thus, performed an exploratory moderation-mediation model to consider these known potential associations and theoretical interactions. We hypothesised that (1) rehabilitation dose mediates the direct effect of the regressors time and pain on the dependent variable function, (2) the direct effect of time and pain on function is moderated by the post-reconstruction period, the effect of pain and time differs in dependence of the different wound healing stages and (3) numerous co-variates, such as pandemic-associated restrictions and pre-injury activity status, contribute to the rehabilitation dose and post-reconstruction functional status.

## Methods

### Design

The present data were assessed directly after the inclusion into a multicentre, single-blind, randomised-controlled, superiority two-arm-trial^22^. Ethical approval has been provided by the Ethics Committee of the Hessen Regional Medical Council (reference approval no. FF 104/2017), and, subsequently, by each centre’s responsible ethics committee. Informed consent was obtained by each participant prior to study enrolment.

When reporting our results, we followed the AGReMA Statement guideline for items to be reported in mediation analyses^23^. All methods were performed in accordance with this guideline and in accordance with the declaration of Helsinki.

### Participants

Adults (age at inclusion from 18 to 35 years) after an acute unilateral ACL rupture and having being passed (< 9 months ago) or scheduled for an arthroscopically assisted, anatomic reconstruction (semitendinosus tendon or semitendinosus-gracilis tendon graft) were included. Only participants engaged in sport (self-reported, any type) prior to the injury and with the aim to return to their previous sporting activity were included.

Exclusion criteria were (1) meniscus lesion > 2 cm, (2) cartilage lesion > ICRS II°, (3) previous musculoskeletal surgery of the uninvolved (contralateral) leg, (4) leg mal-alignment > 5°, (5) multi-ligament injury pattern, (6) post-operative re-injury, (7) acute or chronic inflammation of the musculoskeletal system or muscle soreness and (8) pregnancy.

Potential participants were addressed by personal communication from one of the centre's heads (physicians) during or after a scheduled visit. Screening and recruitment followed a structured informed consent schedule.

### Participants, surgery, and rehabilitation characteristics

After the inclusion, a structured telephone interview was conducted. The type of work (white collar = doing work that needs mental rather than physical effort or blue collar worker = work needing strength or physical skills) and sociodemographic values, such as age, height and weight (body mass index), were asked. The participants, likewise, reported the injury mechanism (contact free, indirect contact, contact) and all (rehabilitation) measures taken between injury and reconstruction^24^ and their pre-injury type(s) of sport and training amount. The athletic level was categorised as either recreational/low-level competitive, semi-professional^25^, or professional.

Further surgery-specific outcomes were retrieved from the previously pseudonymised surgery report: graft type (semitendinosus or semitendinosus-gracilis), tendon folding (from three times up to eight times), tendon diameter [mm] and date of the surgery. From the time between surgery and questionnaire completion, the potential phases (wound healing/rehabilitation) were calculated: initial: 0–2 weeks, early: 2 up to 6 weeks, mid-1: up to 12 weeks, mid-2: up to 26 weeks, late: > 6 months. The wound healing phases acted as moderators. The individual medically prescribed rehabilitation followed a stepwise function-based periodisation and progression^11,13^. Basically, after the diminishing of joint swelling and pain reduction, restoring the knee range of motion was followed by a function-based progression to strengthening and neuromuscular motor control training. Due to local and health-assurance differences, minor between-participant differences regarding the exact design and structuring of the rehabilitation measures may exist.

### Questionnaires

All questionnaires were completed online at www.soscisurvey.de, using the participants’ pseudonym only. The validated German versions of the questionnaires were used.

The survey consisted of the following outcomes (measured by the respective questionnaires): activity level (Tegner activity scale), knee function and symptoms [Knee Injury and Osteoarthritis Outcome Score (KOOS), subscale sport (SPORT), pain (PAIN) and activities of all daily living (ADL)], injury history (free text) and kinesiophobia (Tampa Scale of Kinesiophobia, TSK). The latter two acted as variables to describe the sample.

The Tegner activity scale was included into the model as a co-variate. It contains a 0–10 point Likert scale to assess a participant’s activity level, scaled from low-level daily living activity to high-level competitive sports: scoring is from 0 (= low level activity regarding knee loading), up to and including 10 (= highest possible level of activity regarding knee loading). The KOOS’ subscales are three of the five KOOS subdomains, each item and must be scored from 0 to 4. The PAIN sum score, included as independent variable is calculated based on 9 single items, ADL on 17 and SPORT on 5. The ADL and SPORT subscales were our dependent variables. The TSK uses 11 items across the domain “fear of movement/(re-)injury”: it is scored using a 4-point Likert scale. Internal consistency and structural validity were found to be sufficient^26^.

The training protocol consisted of: therapy type, therapy frequency [times per week], rehabilitation dose [minutes per week], total dose since reconstruction [minutes] and mean perceived exhaustion (Borg scale ratings) [points]. The rehabilitation dose acted as mediator.

The time since reconstruction [days] and the presence or absence of COVID-19-associated restrictions (Co-variate), dichotomised as pre-restriction or during restriction, were calculated based on the date the online survey was filled in.

### Temporal structure and model building

The temporal structure of the variables assessment was: surgery report outcomes were followed by the structured interviews at inclusion, which, in turn, were followed by the questionnaires.

The reconstruction-specific outcomes were retrospectively retrieved from the surgery report, while the structured interview was performed to assess the sociodemographic (also retrospectively) as well as the injury mechanisms and pre-injury training amounts and levels.

The independent variables were the time since surgery and the pain intensity at the questionnaire completion. The mediator was the rehabilitation training volume. The dependent variables were the KOOS SPORT sum score (model 1) and the KOOS ADL sum score (model 2). Co-variates were age, sex/gender, body mass index, time between injury and reconstruction, kinesiophobia, pre- or during COVID-associated restrictions and the pre-injury Tegner activity scale. As moderators, the potential phases (wound healing/rehabilitation) were included in the models.

Temporarily, the independent variable and mediator were assessed retrospectively, while the dependent variable was assessed as the current status; all three were assessed at questionnaire completion. The co-variates were collected retrospectively. The time and pandemic-associated restrictions were retrieved calendrically. Thus, the order of the assessment does comply with the hypothesised order of the mediated moderation model.

### Statistics

Data were subsequently analysed by mediated moderation regression analyses. We used a macro developed and provided by Hayes (PROCESS for SPSS version 3.5.3, model 8^27^). The outcome variables were the KOOS scores, the independent variable was time since reconstruction, the suggested mediator (m) variable was the amount of rehabilitation measures and the (categorised) time-dependent rehabilitation phases were the moderators.

Total, direct, indirect (mediated) and conditionally direct (moderated) effects were calculated for each dependent variable. Sobel testing and 95% confidence interval bias corrected bootstrapping (number of sub-samples = 10,000) were conducted for indirect effect determination. To build (forward/inclusion) the models, R^2^ estimates were adopted.

All statistical calculations were conducted using SPSS 25 (IBM Corporation, New York, NY, USA). Statistical procedures were executed after the examination of the underlying assumptions (visual examination of the scatter plots and by the 1-sample Kolmogorov Smirnov test (raw values and unstandardized residuals), linearity by visual examination of the scatter plots, homoscedasticity, auto-correlation and negligible multicollinearity). The a priori level of significance was set at 5% for all statistical analyses.

### Ethical approval

Ethical approval has been provided by the Ethics Committee of the Hessen Regional Medical Council (reference approval no. FF 104/2017), and, subsequently, by each centre’s responsible ethics committee.

### Informed consent

Yes. Each participant signed informed consent prior to study enrolment.

## Results

In total, 222 persons were recruited and included. During the study conduction, 9 withdrew their consent (due to time constraints: n = 6, or no reason provided: n = 3). Missing values of the dependent variable occurred in 10 participants; these participants were excluded from the present analysis. Data from 203 participants were finally modelled.

The sociodemographic, sport-, injury-, surgery- and pandemic-specific characteristics of the study sample are displayed in Tables 1 (categorical data) and 2 (interval scaled data). We included more males than females and more white- than blue-collar workers. Only a minor share of the sample were at least semi-professional athletes, nevertheless, the sample was highly active both before the injury and as rehabilitation measures; displayed high Tegner activity scores. In addition, most of the injuries were found to have occurred without direct contact with an opponent.

**Table 1:** Numeric and percentage distributions of all categorical sociodemographic, sport-, injury-, surgery- and pandemic-specific characteristics of the study sample.

Domain	Outcome	Value	n	%
Sociodemographic	Sex/gender	Female	83	41
		Male	120	59
		Diverse or non-binary	0	0
	Type of work	White collar	152	75
		Blue collar	51	25
Sport	Athletic level	Recreational/low-level competitive	189	93
		Semi-professional or professional	15	7
	Tegner activity level pre-injury	3	13	6
		4	48	24
		5	12	6
		6	37	18
		7	74	36
		8	4	2
		9	13	6
		10	3	2
Injury	Injury mechanism	Contact free	151	74
		Indirect contact	35	17
		Direct contact	17	9
Surgery	Graft type	Semitendinosus	67	53
		Semitendinosus-gracilis	60	47
	Tendon folding	3x	6	3
		4x	160	83
		5x	3	1
		6x	17	9
		7x	6	3
		8x	3	1
Rehabilitation	Phases/stages (wound healing)	Initial: 0–2 weeks	60	29
		Early: 2 up to 6 weeks	65	32
		Up to 12 weeks	26	13
		Up to 26 weeks	33	16
		> 6 months	20	10
Covid-19		Pre-restriction	128	63
		During restriction	75	37

*n* numbers, *%* percentage share.

**Table 2:** Mean (median, where urgent) and standard deviations of all interval (and pseudo interval) scaled sociodemographic, sport-, injury-, surgery- and rehabilitation measures, including specific characteristics as well as the self-report functional outcomes of the study sample, *TSK* Tampa Scale of Kinesiophobia.

Domain	Outcome	Mean	Standard deviation
Sociodemographic	Body mass index (kg/m^2^)	25	4.1
	Age (years)	26	5.0
Sport	Training amount pre-injury (minutes/week)	299	251
Surgery	Time between injury and reconstruction (days)	122 (median 56)	269
	Tendon diameter (mm)	8.1	0.85
	Time since reconstruction (days)	61 (median 33)	67
Rehabilitation	Therapy frequency (times per week)	2.5	2.0
	Rehabilitation dose (minutes per week)	149	151
	Total dose since reconstruction (minutes)	1115	1562
	Mean Borg scale (points)	8.5	3.2
Self-report outcomes	KOOS SPORT (points)	37	33
	KOOS PAIN (points)	65	22
	KOOS ADL (points)	71	25
	Kinesiophobia (TSK sum score) (points)	24	4.6

**Figure 1:**
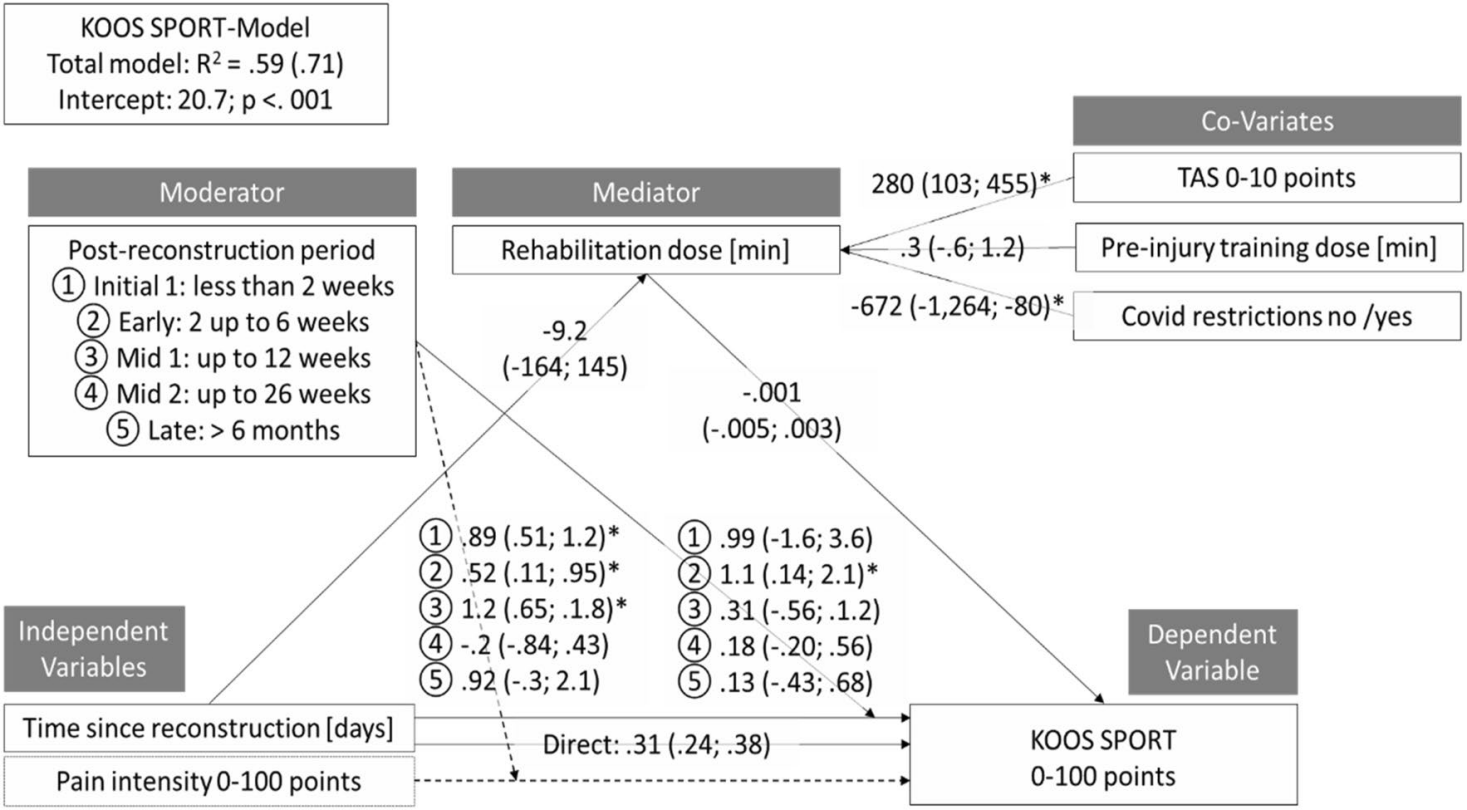
Moderated mediation model outcomes for the dependent variable KOOS SPORT. Coefficients and their confidence intervals (lower limit; upper limit) are displayed for direct, conditional direct, total and indirect effects of pain and time since reconstruction. The moderation effects of the different rehabilitation phases on the regression of pain intensity (left column) and time since reconstruction (right column) on KOOS SPORT are displayed, likewise. Asterisks (*) display a significant interaction. *TAS* Tegner activity scale, *min* minutes, *R*^*2*^ variance explanation without (and, in brackets, variance explanation with) pain intensity as a sub-model.

**Figure 2:**
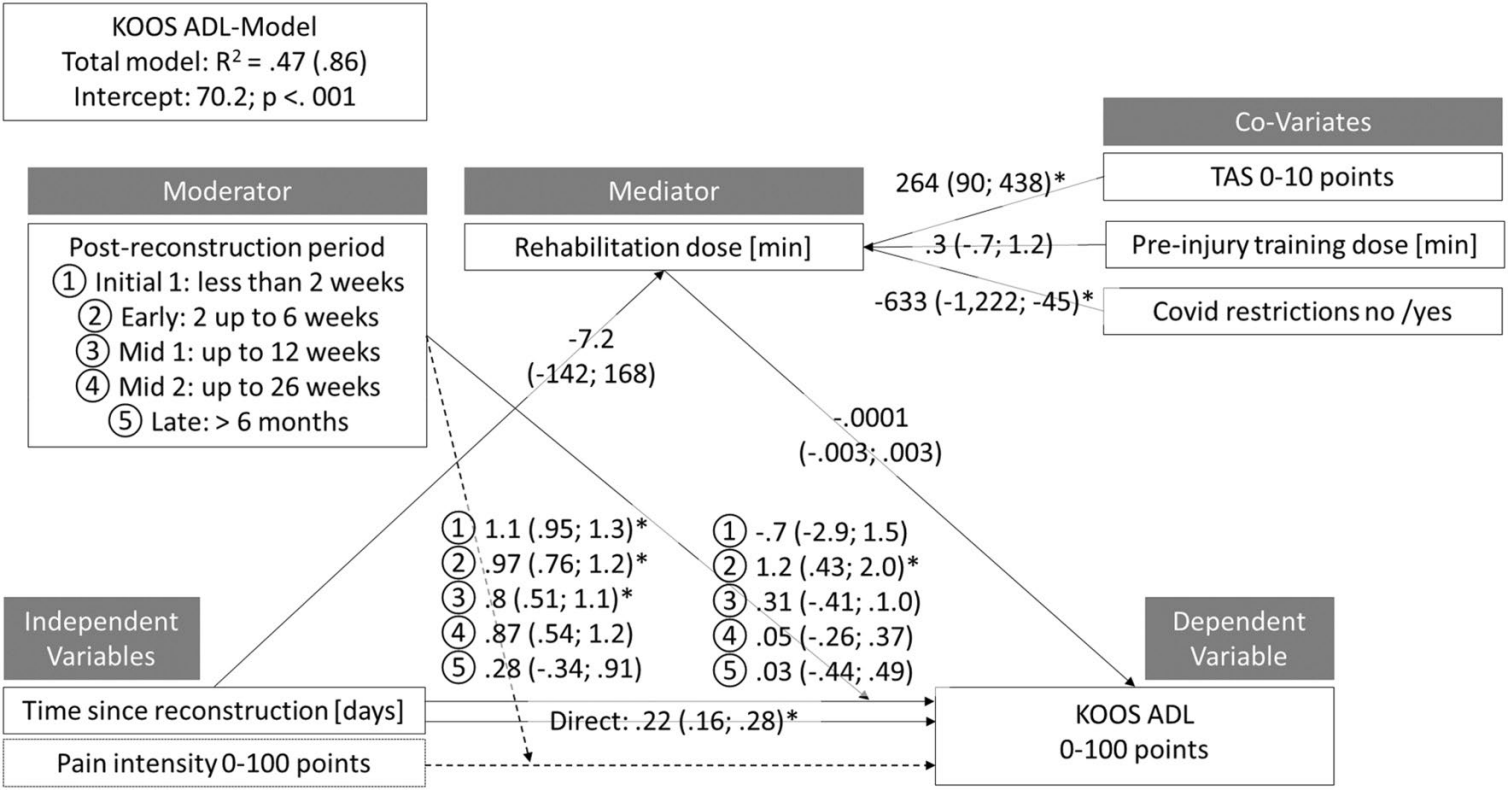
Moderated mediation model outcomes for the dependent variable KOOS ADL. Coefficients and their confidence intervals (lower limit; upper limit) are displayed for direct, conditional direct, total and indirect effects of pain and time since reconstruction. The moderation effects of the different rehabilitation phases on the regression of pain intensity (left column) and time since reconstruction (right column) on KOOS ADL are displayed, likewise. Asterisks (*) display a significant interaction. *TAS* Tegner activity scale, *min* minutes, *R*^*2*^ variance explanation without (and, in brackets, variance explanation with) pain intensity as a sub-model.

The final models, one for the dependent variable sum score for KOOS SPORT and one for the corresponding KOOS ADL-sum-score, are displayed in Figs. 1 and 2. The significant direct positive effect of the time passed since reconstruction on the KOOS-values is mostly attributed to (moderated by) the early rehabilitation phase. The direct effect of pain on KOOS occurs in the initial, early and mid-1-rehabilitation phases. The rehabilitation dose is strongly increased when higher Tegner scores are present; the COVID-19-associated restrictions decreased these training volumes.

## Discussion

The self-report function after autologous hamstring graft ACL-reconstruction is influenced by several factors. Rehabilitation had no significant mediator role in the way how time since reconstruction or pain intensity influence self-reported function. In the initial rehabilitation stage, pain was the strongest regressor of the dependent variable to self-report function. In the following early stage, time since reconstruction becomes a further major regressor to the self-report function. Starting with the afterwards commencing mid-phases of the rehabilitation, the self–report function is not explicitly impacted by one or more independent variables or mediators any more. The amount of rehabilitation training itself is strongly affected by COVID-19-associated restrictions and by the pre-injury activity scale. Other hypothesised co-variates, such as sex/gender or age, were not found to be moderators or mediators in the time or pain, rehabilitation dose and self-report function triangle. Our hypothesis (1), suggesting that the rehabilitation dose mediates the direct effect of the regressors time and pain on the dependent variable function, must, thus, be rejected because the rehabilitation dose did not mediate the effect of time on function. Hypothesis (2), suggesting that the effect of pain and time differs in dependence of the different wound healing stages was verified since the contributors to function are not unique in the different wound healing stages/rehabilitation phases. The same is true for hypothesis (3), that numerous secondary co-variates, such as pandemic-associated restrictions and pre-injury activity status, would contribute to the rehabilitation dose and post-reconstruction functional status: we found a few secondary contributors to the rehabilitation measures and port-reconstruction functional status.

The association of function and pain intensity during performance is in accordance with current comparable evidence^16^. The time passed since reconstruction was only a relevant predictor of function during the early rehabilitation phase. This provides another hint that performing one single assessment at the hypothetical end of the RTS process is not goal achieving^28^. Multiple repetitive measurements, aimed to monitor and verify the course of the RTS process, are more promising^28^. Such a repetitive measurement approach over time considers both time and (functional) status factors and was found to be feasible (also including questionnaires) in an athletic RTS setting^29^.

During the different rehabilitation stages, different factors are of interacting relevance^11,13^. Initially, restoring the passive knee extension range of motion is aimed for (after the diminishing of joint swelling and pain reduction); this seems to be in accordance with our finding of (at the initial phase) the major contributor pain (to function). The next rehabilitation phase (the early phase), usually 2–4 weeks post-surgery, includes a function-based progression to strengthening and neuromuscular control training. Here, the inter-individual contributors (i.e. time since reconstruction) increase, not only pain seems to be decisive for self-report function, any more. Starting with the progressive strengthening/neuromuscular control phase, the contribution of time, again, decreases. The contribution of pain is, lastly, not present any more in the last (advanced activity) phase.

Knowing the contributors to functional abilities (estimate/coefficient) and their interactions is helpful for the function-based/deficit-oriented rating and management of rehabilitation and RTS strategies. Improving or even restoring functional abilities and, thus, decreasing the identified deficit, may consequently lead to a decrease in the subsequent injury risk^10^. Such functional abilities also includes those measured at a low-threshold self-report questionnaire level (such as the KOOS). The KOOS-SPORT-sub-score may be re-injury-predictive^6^, thus, improving factors (functions), which are mirrored by this outcome, may consequently be helpful in re-injury prevention after ACL-reconstruction.

We only included participants after an isolated ACL rupture with a subsequent reconstruction using a hamstring graft. Potential contributors (or even confounding variables) to function, such as concomitant injuries and other graft types^17,18,21,30–32^, were, thus, not included. The present sample is, consequently, representative for a young and physically active sample after hamstring graft reconstruction.

The association of function and pain intensity during performance^16^ is not surprising, but this must also be considered when function should be rated more holistically, i.e. in dependence of the rehabilitation/wound healing phase. Here, the interactive description of the various factors must be considered as a strength of this analysis. However, we only reported associations and no experimentally derived effects, therefore, this must be considered as a major limitation of this analysis. Although statistically sufficiently powered, the relatively small sample size might lead to a certain overfitting and, thus, be a source of bias for the transferability to the underlying population.

When considering all potential contributors and their interactions, we found no clear mediating contribution of the rehabilitation dose. Although a clear dose–response relationship has not been described in the literature (on rehabilitation after ACL reconstruction), higher scheduled rehabilitation adherence/compliance may lead to better functional outcomes^33,34^. In the present sample, the dose of rehabilitation measures was impacted by the pre-injury Tegner activity scale and COVID-19-associated restrictions. Participants with a higher Tegner activity scale may be more compliant than those with a lower scale. Concerning the outcome, the KOOS may not be sensitive enough in the later stages when compared to the more late-stage-associated functional scores, such as the ACL Return to Sports Injury Scale (RSI-ACL) or even objective functional outcomes. In contrast, the KOOS can, unlike objective functional tests, be applied very early after the reconstruction and may, thus, be able to mirror function during the most phases after the reconstruction. Among the most selected self-reported functional outcomes accessible at an early stage, the KOOS outperformed the International Knee Documentation Committee Subjective Knee Form (IKDC)^35^. Another potential consequence of only applying a self-reported outcome has recently been elegantly shown in a re-injury predictive analysis: patients at RTS who passed a battery of functional tests and showed low knee-related confidence were at lower risk for a re-injury than those who were both confident and met all RTS criteria^36^. A certain overrating of one's own knee-related abilities can, thus, also be re-injury-risk enhancing rather than preventing. Including further potential contributors to self-reported function such as residual laxity might have provided further variance explanation. The time categories we set followed potential wound healing phases but are, of course, somewhat arbitrary and could also be set differently.

The finding of the pandemic-restrictions effect on rehabilitation measures provides another hint on the relevance of considering pre-versus during (versus post-, if applicable) COVID-19-associated restrictions (or even the specific measures) as a categorising variable. In orthopaedic settings, this may impact both surgery^37^ and rehabilitation measures. The contact restrictions lead to an increase in the importance of (supervised and controlled) home-based and/or telemedicine measures, as well as in the (late stages of the) rehabilitation. This is one of the advantages of the trial of which the inclusion visit data are presented here^22^.

When known potential contributors and confounders, such as in the present analyses, are considered, gender/sex and age were not significant contributors to the self-report function. This is somewhat in contrast with major parts of the literature^15^. Whether this is attributable to the somewhat (in terms of physical activity and age) homogeneous sample, or if these contributors are not significantly relevant in the underlying sample when all relevant interacting factors are considered, can only be speculated.

As highlighted in the limitations, we only calculated cross-sectional associations and no longitudinal effects. To prove (or disprove) our findings, the associations found should be reproduced in a longitudinal setting in future study.

## Conclusion

Numerous factors, such as pain, the time since reconstruction, different wound healing stages and rehabilitation phases, contribute to the course of self-reported functional abilities after ACL-reconstruction. The amount of rehabilitation measures required is further impacted by the participant’s pre-injury activity and COVID-19-associated restrictions. Knowing these factors and also knowing their nested contribution value is helpful for the management of deficit-oriented function-based rehabilitation and individualised RTS strategies. When self-report function is rated, the rehabilitation phases (early, mid, late), the potentially COVID-19-associated rehabilitation limitations, and pain intensity should be considered in decision-making. Focussing on the value of the self-report function only may, consequently, not be sufficient to rate bias-free function.

## Data Availability

The datasets used and/or analyzed during the current study available from the corresponding author on reasonable request.
